# Integrated analysis for drug repositioning in migraine using genetic evidence and claims database

**DOI:** 10.3389/fdata.2025.1677167

**Published:** 2025-11-21

**Authors:** Shoichiro Inokuchi, Takumi Tajima

**Affiliations:** Real World Evidence Division, Pharmaceutical Division, JMDC Inc., Tokyo, Japan

**Keywords:** migraine, drug repositioning, human genetics, routinely collected health data, drug database

## Abstract

**Introduction:**

Migraine is a prevalent neurological disorder with a substantial socioeconomic burden, underscoring the need for continued identification of therapeutic targets. Given the significant role of genetic factors in migraine pathogenesis, a genetic-based approach is considered effective for identifying potential therapeutic targets. This study aimed to identify candidate treatments for migraine by integrating genome-wide association study (GWAS) data, perturbagen profiles, and a large-scale claims database.

**Methods:**

We used published GWAS data to impute disease-specific gene expression profiles using a transcriptome-wide association study approach. The imputed gene signatures were cross-referenced with perturbagen signatures from the LINCS Connectivity Map to identify candidate compounds capable of reversing the disease-associated gene expression. A real-world claims database was subsequently utilized to assess the clinical efficacy of the identified perturbagens on acute migraine, employing a cohort study design and mixed-effects log-linear models with the frequency of prescribed acute migraine medications as the outcome.

**Results:**

Eighteen approved drugs were identified as candidate therapeutics based on the perturbagen profiles. Real-world analysis using the claims database demonstrated potential inhibitory effects of metformin (relative risk [RR]: 0.81; 95% confidence interval [CI]: 0.77–0.86), statins (RR: 0.94; 95% CI: 0.92–0.96), thiazolidines (RR: 0.84; 95% CI: 0.73–0.97), and angiotensin receptor neprilysin inhibitors (RR: 0.69; 95% CI: 0.61–0.77) on migraine attacks.

**Conclusion:**

This multidisciplinary approach highlights a cost-effective framework for drug repositioning for migraine treatment by integrating genetic, pharmacological, and real-world clinical database.

## 1 Introduction

Migraine is a common neurological disorder that affects approximately 15% of the global population and is characterized by recurrent headache episodes, often accompanied by concomitant manifestations, including aura (GBD 2016 Headache Collaborators, 2018). Its prevalence is the highest among women aged 35–39 years. This disorder imposes a considerable socioeconomic burden, as acute migraine attacks can significantly disrupt daily activities, including educational and occupational responsibilities. Despite advancements in treatment strategies, migraine remains the second leading cause of disability-adjusted life-years among patients with neurological conditions, highlighting the profound disability and economic impact associated with this disease (GBD 2016 Neurology Collaborators, 2019). These challenges underscore the need for continued efforts to identify novel therapeutic targets for migraine management.

In drug discovery, effective screening is essential to successfully identify potential candidate drugs, and drug discovery and repositioning based on genetic findings have proven useful (Nelson et al., 2015; King et al., 2019; Reay and Cairns, 2021). These methodologies include the identification of disease-associated target genes based on genetic variants, selection of drug repositioning candidates informed by risk gene sets and known drug targets (Sakaue and Okada, 2019), approach based on transcriptome-wide association study (TWAS) (Konuma et al., 2021; Namba et al., 2022; Wu et al., 2022), and Mendelian randomization analyses (Hemani et al., 2018), as well as bioinformatic and deep-learning frameworks for predicting therapeutic targets and drug responses, including reference-free transcriptomic and graph neural-network approaches that highlight the growing role of data-driven methods in drug discovery (Eralp and Sefer, 2024; Sefer, 2025). Given that migraine has substantial genetic attributes, with an estimated heritability of 42% (Polderman et al., 2015), leveraging genetic information may facilitate the identification of therapeutic targets and support efforts in drug discovery and repositioning. Additionally, recent studies have demonstrated the utility of genetic evidence in conjunction with clinical databases (Wu et al., 2022).

To identify potentially effective treatments for migraine, we performed an integrative analysis using the results from a previously published genome-wide association study (GWAS) meta-analysis (Choquet et al., 2021) and a gene perturbation database, followed by clinical validation using a routinely collected claims database. This approach not only enhances the robustness of the findings by incorporating multiple data sources but also provides a cost- and time-efficient strategy for drug repositioning.

## 2 Materials and methods

### 2.1 Data source

This study used publicly available data from a previously published GWAS meta-analysis (Choquet et al., 2021). This meta-analysis incorporated data from multiple ancestries, encompassing a total of 554,569 individuals (including 28,852 migraine cases) from the Genetic Epidemiology Research in Adult Health and Aging (GERA) cohort and UK Biobank (UKB) cohorts. Only aggregated data were used, and no individual-level data were analyzed.

For perturbagen profiles, the Library of Integrated Network-based Cellular Signatures (LINCS) Connectivity Map (CMap) L1000 dataset was employed (Konuma et al., 2021; Wu et al., 2022). Level 5 data (moderated Z-scores) were downloaded from the Gene Expression Omnibus (GEO) database (accession numbers GSE92742 and GSE70138).

To evaluate the potential effectiveness of candidate drugs for migraine, as suggested by genetic evidence and perturbagen profiles, we analyzed data from the JMDC Claims Database (Nagai et al., 2021), which is derived from routine clinical practice. This anonymized database contains insurance claims data from health insurance societies and includes information from approximately 20 million individuals. The dataset encompasses diagnostic codes, prescription records, clinical procedures, and health checkup results. Importantly, the health insurance societies represented in this database do not include elderly individuals; thus, data from individuals aged 75 years or older were not included.

As the study used anonymized secondary data, ethical approval was not required in accordance with the local regulations. This study was conducted in accordance with the principles of the Declaration of Helsinki.

### 2.2 Imputation of disease specific gene signature

To estimate tissue-specific gene expression signatures associated with migraine using GWAS summary statistics, we conducted a TWAS using the FOCUS method (Mancuso et al., 2019; Lu et al., 2022). FOCUS employs a Bayesian approach that integrates GWAS summary statistics and gene expression weights derived from expression quantitative trait loci (eQTL) data to infer disease-specific gene expression profiles. eQTL reference data were obtained from the Genotype-Tissue Expression (GTEx) project (version 8) (Gamazon et al., 2015; Barbeira et al., 2018, 2021). In FOCUS, the TWAS z-score is estimated using the following formula:


$$\begin{array}{l} Z_{TWAS}=\frac{W^{T}X}{\sqrt{W^{T}VW}} \end{array}$$

Where *W* is the eQTL weight matrix, *X* is the vector of GWAS *z*-scores, and *V* is the SNP reference linkage disequilibrium (LD) matrix. Tissues used for estimating disease-specific signatures were selected based on tissue-specific enrichment analysis using the deTS algorithm (Pei et al., 2019), which relies on disease-associated genes identified via the Multi-marker Analysis of GenoMic Annotation (MAGMA) method (de Leeuw et al., 2015) (*p* < 0.05). Additionally, considering the neurological involvement in migraine, the top three nervous system tissues were included, along with whole blood, to account for the potential contribution of neuroinflammation.

### 2.3 Identification of candidate perturbagens

We integrated tissue-specific TWAS data derived from FOCUS with gene expression profiles from the LINCS CMap L1000 library to identify perturbagens capable of normalizing disease-associated gene expression signatures. To this end, we examined the inverse correlations between disease-specific tissue gene expression signatures and perturbagen-induced gene expression profiles, using the LINCS L1000 dataset. From each tissue selected through tissue-specific enrichment analysis, we extracted the top 10% of the genes with the highest absolute TWAS *z*-scores. Spearman's rank correlation coefficients were calculated for these genes to assess the negative relationship between TWAS-derived expression profiles and LINCS L1000 perturbagen-induced profiles (Konuma et al., 2021; Wu et al., 2022).


$$\begin{array}{l} Spearman^{\prime }s\rho =\frac{cov\left(R\left(Z_{TWAS}\right),R\left(Z_{LINCS}\right)\right)}{\sigma_{R\left(Z_{TWAS}\right)}\sigma_{R\left(Z_{LINCS}\right)}} \end{array}$$

*Z*_*LINCS*_ represents the *z*-score of the change in gene expression induced by the perturbagen, *R*(.) denotes the rank, and σ indicates the standard deviations of the rank variable. This generated a correlation coefficient for each tissue perturbagen pair. Perturbagen with a one-sided *p*-value < 1 × 10^−4^ were considered candidate therapeutics. This approach is hereafter referred to as the “Spearman-based method.”

In parallel, we used the LINCS SigCom platform to identify the candidate compounds. SigCom is a web-based platform designed to identify mimicker or reverser perturbagens based on upregulated and downregulated gene inputs and to associate gene signatures with similar cell types or diseases (Evangelista et al., 2022). We submitted the top 150 upregulated and bottom 150 downregulated genes derived from the tissue-specific TWAS results to SigCom. The top 200 reverser perturbagens were identified using the Mann-Whitney U test. This approach is hereafter referred to as the “SigCom-based method.”

### 2.4 Validation using a claims database: study population

To validate the suggested drugs for migraine treatment identified through GWAS data and perturbagen profiles, a cohort study was conducted using the JMDC Claims database. The database includes claims records for diagnoses, prescriptions, and medical procedures from routine clinical practice. Eligible patients were included based on the following criteria: (1) diagnosis of migraine (International Statistical Classification of Diseases and Related Health Problems 10th Revision [ICD-10]: G43) between September 2018 and August 2023; (2) at least one prescription for migraine-specific medications (Supplementary Table 1); (3) continuous enrollment for ≥180 days; (4) no history of ischemic heart disease (ICD-10: I20–I25), stroke (ICD-10: I60–I69), or transient ischemic attack (ICD-10: G45); and (5) a follow-up period of at least 1 d. The first diagnosis date was designated as the index date (Day 0). The follow-up continued until death, emigration of the database, or occurrence of heart failure, stroke, or transient ischemic attack, whichever came first.

### 2.5 Validation using a claims database: variables

As migraine attacks could not be directly obtained from the database, the number of acute migraine drugs, including triptans, ergotamine, and ditans (Supplementary Table 1), were used as surrogate outcomes. We did not consider paracetamol, non-steroidal anti-inflammatory drugs, antipsychotics, or antiemetics because these medications can be prescribed for any other condition, thereby exhibiting low specificity.

Drugs suggested by both methods (i.e., *P*_*spearman*_∩*P*_*sigcom*_; where *P*_*spearman*_, *P*_*sigcom*_ denotes perturbagens identified by Spearman- and SigCom-based methods, respectively) were considered as exposure. The exposures were treated as time-varying variables. Covariates were selected based on the treatment strategy for migraine and potential risk factors of acute migraine (Amiri et al., 2021). Potential confounders for each specific drug exposure were also considered to adjust for patient characteristics associated with the use of each drug of interest (Supplementary Table 2). Exposure status and covariates were defined based on the presence or absence of each diagnosis or medication within the covariate assessment window at each time point.

### 2.6 Validation using a claims database: statistical analysis

To evaluate the treatment effect on migraine attacks, the number of prescribed acute migraine medications was used as an outcome measure. A rolling 1-year time window was applied to account for time-varying variables, and the effect of each candidate drug identified through genetic analysis was evaluated using a mixed-effect log-linear model. The model is specified as follows.


(3) $$\ln(Y_{ij}+1)=\beta_{0}+\alpha t+\sum_{k=1}^{C}\beta_{k}X_{kij}+\gamma_{i}+\ln time_{ij}+\epsilon_{ij}$$

where βs are the fixed effects, *Y*_*ij*_ denotes the number of prescribed acute migraine medications for patient *i* at time window *j*, *t* is a binary indicator of exposure drug, *C* represents the number of covariates, *X*_*kij*_ is the value of the *k*-th covariate for patient *i* at time window *j*, γ_*i*_ is the random effect for patient *i*, *time*_*ij*_ denotes the duration of the outcome evaluation window (i.e., included as an offset term), and ϵ_*ij*_ is the random error term. The exponential coefficient exp(α) was interpreted as the multiplicative effect of the exposure drug on the frequency of prescribed acute migraine medications. The analysis using the claims database was informed by RECORD checklist (Benchimol et al., 2015) (Supplementary materials).

Data preprocessing was performed using Python version 3.12.2, and statistical analyses were performed using R version 4.3.3.

## 3 Results

### 3.1 Identification of candidate perturbagens that normalize migraine-specific gene expression

**Figure 1:**
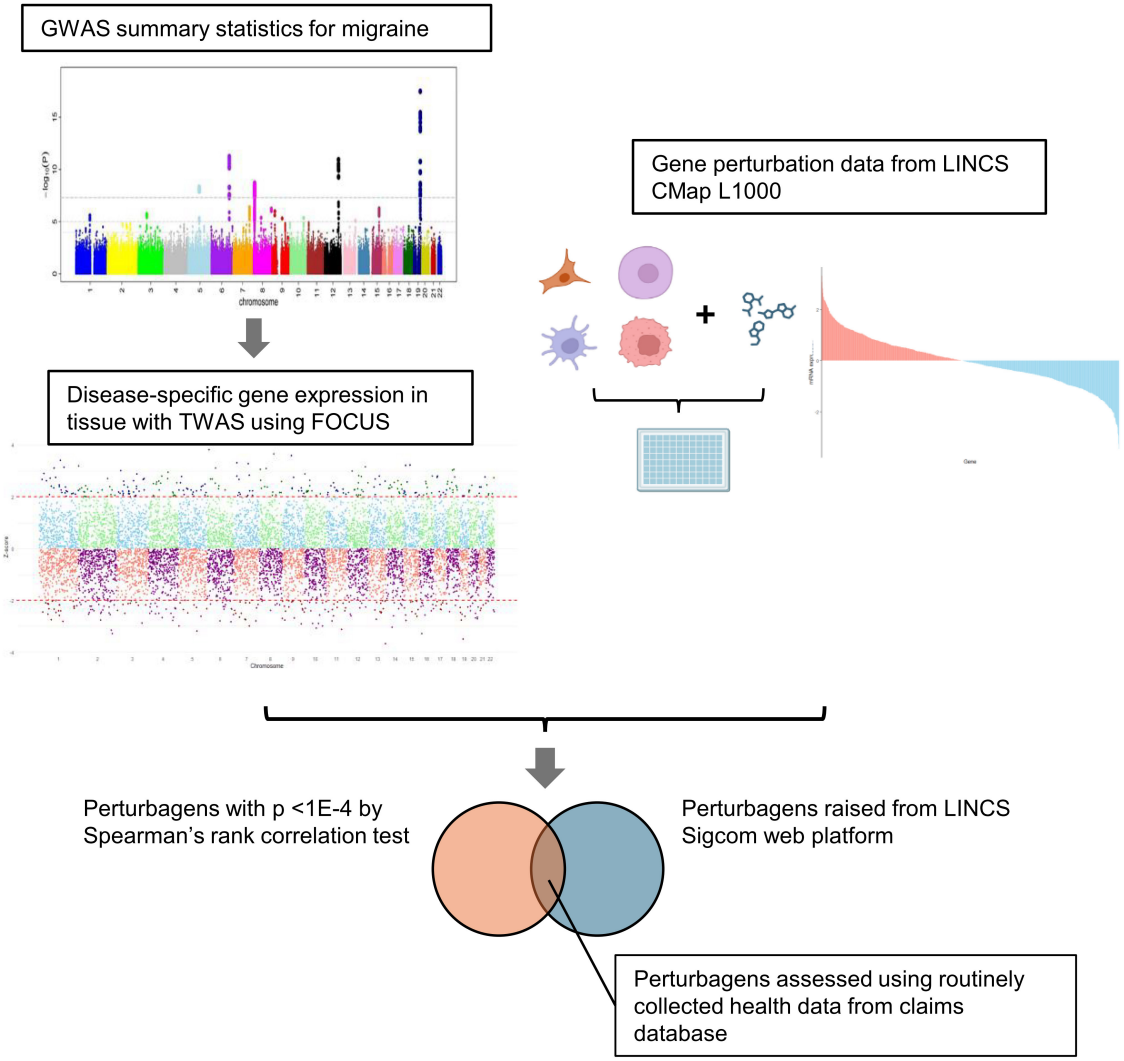
Schema of the study. Disease-associated gene signatures were imputed using GWAS summary statistics and FOCUS, which is a Bayesian TWAS framework. The imputed gene signatures were cross-evaluated with gene perturbation data from LINCS CMap L1000 using two approaches: Spearman's rank correlation test and SigCom platform. Common perturbagens identified using both methods were extracted and validated using a claims database.

**Figure 2:**
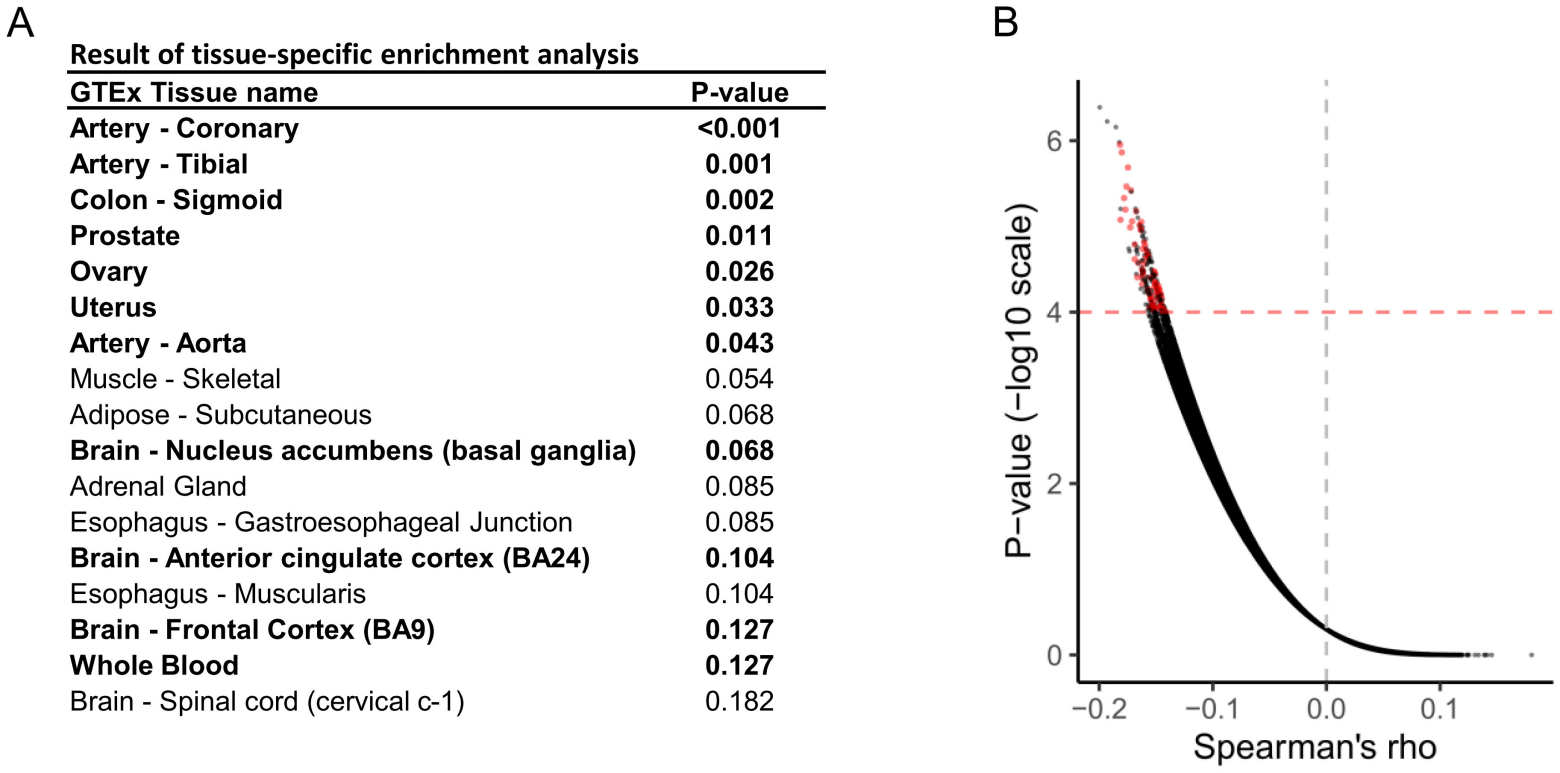
Identification of potential therapeutic medications for migraine. **(A)** Results of tissue-specific enrichment analysis of migraine. **(B)** Volcano plot for the Spearman's rank correlation test, which evaluated the inverse correlation between disease-specific gene signatures and gene perturbation data obtained from LINCS CMap L1000. Each dot represents a perturbagen-cell pair with a one-sided *p*-value. Red dots indicate perturbagens with therapeutic potential identified using the SigCom platform.

The overall scheme is illustrated in Figure 1. We used publicly available data from the GWAS meta-analysis study (Choquet et al., 2021). First, we performed tissue-specific enrichment analysis using the deTS algorithm (Pei et al., 2019) based on disease-associated genes identified using the MAGMA method (de Leeuw et al., 2015). Based on tissue-specific enrichment analysis, tissues with *p* < 0.05, along with whole blood and the three most relevant neuronal tissues, were selected for the subsequent TWAS analysis using FOCUS (Figure 2A). FOCUS is a Bayesian estimation algorithm that is used to impute disease-specific tissue signatures based on GWAS summary statistics and eQTL weights. Next, we evaluated the imputed disease-specific signature against perturbagen profiles from LINCS CMap L1000 library data (Wu et al., 2022). Perturbagens that demonstrate inverse associations with disease signatures are considered as potential therapeutic candidates. Additionally, we used the SigCom framework, a web-based platform that identifies signatures that mimic or reverse perturbagens based on input gene lists (Evangelista et al., 2022). The top 150 upregulated and downregulated genes identified by TWAS analysis were used as inputs, and 200 reversers were considered as candidate perturbagens capable of normalizing migraine-specific gene expression profiles. Among the 84 perturbagens identified from both approaches, 18 approved drugs were considered potential drugs that normalized migraine-specific gene expression (Figure 2B; Supplementary Table 3).

### 3.2 Clinical validation of identified drugs using a routinely collected claims database

**Figure 3:**
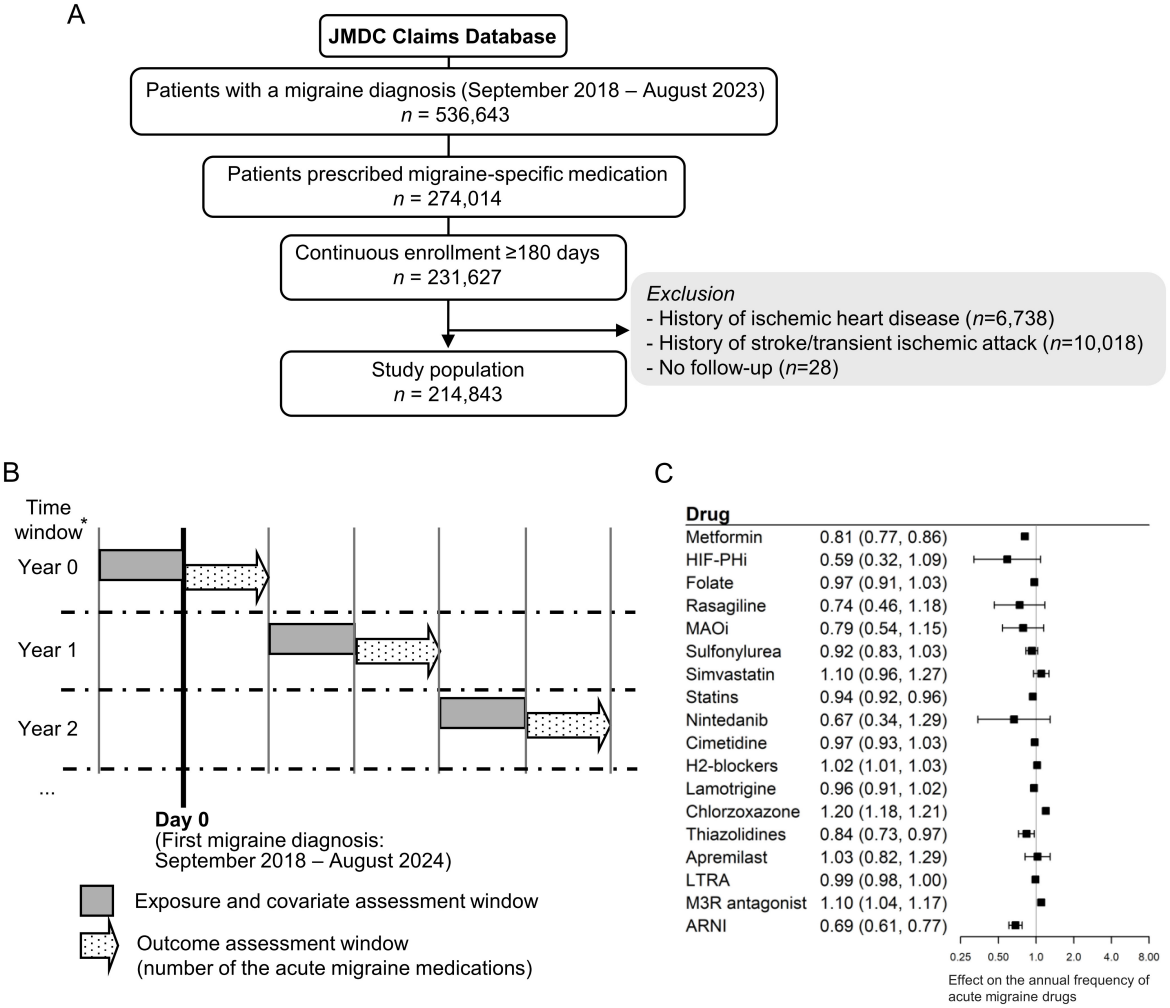
Clinical validation of identified candidate drugs using a large claims database. **(A)** Flow diagram illustrating the clinical validation using the claims database. **(B)** Study design for clinical validation using a claims database. *Patients were followed up until the earliest exit from the database, death, or the onset of ischemic heart disease, stroke, or transient ischemic attack. **(C)** Forest plot of clinical validation. A separate mixed-effects log-linear model estimating the annual frequency of acute migraine drugs (triptans, ergotamine, and ditans) was developed for each exposure drug. The exponential coefficient of the exposure term is shown, representing the multiplicative effect on the frequency of prescribed acute migraine medications. MAOi, Monoamine oxidase inhibitor; LTRA, Leukotriene receptor antagonist; M3R, M3 muscarinic acetylcholine receptor; ARNI, Angiotensin receptor neprilysin inhibitor.

For claims data analysis, we used the JMDC Claims Database (Nagai et al., 2021), which comprises anonymized insurance claims data for approximately 20 million individuals in Japan. This analysis included 214,843 patients diagnosed with migraine between September 2018 and August 2023 (Figure 3A). The median age was 38 years (interquartile range [IQR]: 26.0–47.0), and 71.1% of the included patients were female. The median duration since the initial diagnosis of migraine in the database was 0 years (IQR: 0.0 to 1.6), and the median follow-up period was 965 days (IQR: 522.0–1,603.0) (Supplementary Table 4). Prescriptions for acute migraine medications were relatively higher in the first year (median 5, IQR 0–40) compared with the overall period (median 2, IQR 0–29). To evaluate the treatment efficacy for migraine attacks, the frequency of prescribed acute migraine medications was used as a measure of outcome. A rolling 1-year time window was applied to account for time-varying medications (Figure 3B), and the effects of 18 candidate drugs were evaluated using a multivariable mixed-effect log-linear model. The results suggested a potential inhibitory effect of metformin (relative risk [RR]: 0.81 [95% confidence interval {CI}, 0.77–0.86]), statins (RR: 0.94 [95% CI: 0.92–0.96]), thiazolidines (RR: 0.84 [95% CI: 0.73–0.97]), and the angiotensin receptor neprilysin inhibitor (ARNI, RR 0.69 [95% CI: 0.61–0.77]) (Figure 3C). Several agents, including nintedanib and hypoxia-inducible factor prolyl hydroxylase inhibitors (HIF-PHi), also showed a trend toward protective effects (RR: 0.59, [95% CI: 0.32–1.09] and RR: 0.67, [95% CI: 0.34–1.29], respectively).

## 4 Discussion

In this study, we used an integrative approach to identify potential therapeutic agents for migraine by leveraging genetic, perturbagen, and real-world claims databases. Using TWAS based on a large-scale GWAS, we imputed migraine-specific gene expression signatures and screened for perturbagens capable of reversing disease signatures using data from the LINCS Connectivity Map. Eighteen approved drugs were identified as candidate agents, including metformin, statins, thiazolidines, and ARNI, which demonstrated significant associations with reduced acute migraine attacks in an analysis using a real-world claims database. The use of routinely collected prescribed data is known to be subject to confounding by indication (Acton et al., 2023). Although residual confounding cannot be ruled out, potential confounding variables were carefully selected, including common confounders such as age, sex, diabetes, and dyslipidemia, as well as exposure-specific confounders (e.g., chronic kidney disease and dialysis for HIF-PHi; rheumatoid arthritis and other rheumatic diseases for folate), to mitigate this bias. These findings support the feasibility and utility of integrating genomic data with pharmacologic perturbation and routinely collected health databases to accelerate drug repositioning efforts for complex neurological diseases such as migraine.

The analysis of GWAS statistics and perturbagen data identified the mammalian target of rapamycin (mTOR) as a potential target for migraine. Among the approved drugs, metformin was selected as a potent mTOR inhibitor (Howell et al., 2017). Metformin, an anti-diabetic agent, has been shown to attenuate neuroinflammation in a mouse model of migraine (Fan et al., 2024), supporting the findings of the study. Despite the lack of significant benefits observed in a previous randomized clinical trial (ClinicalTrials.gov ID: NCT02593097), which was limited by the small sample size, the findings of the present study related to large-scale claims data analysis suggest that metformin may be a promising therapeutic option for migraine, warranting further investigation.

Statins, which are inhibitors of 3-hydroxy-3-methylglutaryl-coenzyme A reductase (HMGCR) and are widely used as lipid-lowering agents, may also influence migraine. Evidence suggests an association between HMGCR expression and the risk of migraine; hence, the use of statins has been proposed to reduce both the development and frequency of attack of migraine (Makhlouf et al., 2025). Although the precise mechanisms by which statins affect migraine pathology are not fully understood, their anti-inflammatory properties, potential to improve endothelial function, and ability to ameliorate metabolic disturbances may contribute to their therapeutic effects. Although the overall use of statins showed a protective association with migraine attacks, simvastatin did not exhibit this effect. Given the previous evidence supporting the efficacy of simvastatin combined with vitamin D (Buettner et al., 2015), examining the differential effects of various statins may provide valuable insights into their potential roles in migraine management.

The current study provides the first evidence suggesting the potential utility of thiazolidines and ARNI as therapeutic interventions for migraine. Thiazolidines, a class of pharmaceutical agents primarily used for the treatment of type 2 diabetes mellitus, exert their effects through the agonistic activity of peroxisome proliferator-activated receptor gamma (PPARγ) (Gamo et al., 2014). PPARγ, a member of the nuclear hormone receptor superfamily, is implicated not only in the pathophysiology of glucose and lipid metabolic dysregulation but has also been reported to exhibit anti-inflammatory and neuroprotective properties (Kapadia et al., 2008). ARNI is a recently approved antihypertensive agent that comprises an angiotensin II receptor blocker (ARB) and neprilysin. The observed effect of ARNI likely reflects mechanisms beyond those mediated by ARB alone, which was already accounted for in the clinical validation model using the claims database. Pro-brain natriuretic peptide has been reported to be associated with migraine, potentially implicating a link between heart failure and migraine (Uzar et al., 2011). This association might suggest that improving heart failure by ARNI could result in the amelioration of migraine pathology. However, to date, the relationship between thiazolidine or ARNI and migraine has not yet been fully explored, necessitating further research to elucidate this potential association.

This study had several limitations. First, although migraine is a genetically significant disorder, this study is based solely on genetic evidence as it does not account for environmental factors that also play a critical role in the pathophysiology of migraine. Second, the perturbagen profiles are primarily derived from cancer cell lines rather than primary cells. This may result in inaccurate estimation of perturbagen effects in disease-relevant tissues, potentially leading to the omission of therapeutically relevant compounds. To address this limitation, incorporating datasets generated from primary cells or organoid systems may enhance the accuracy and applicability of the method used in the study. Third, for clinical validation, we used routinely collected health data, which lacked detailed migraine-specific clinical information such as migraine subtype and severity. This limitation introduces a potential for residual confounding. Additionally, it necessitated the use of acute migraine medication prescriptions as a surrogate measure of migraine burden. Prescribing patterns may be influenced by both patient behaviors, physician practice, and comorbidity profiles. Although baseline variables including comorbidities were adjusted for in the study, residual confounding may remain, potentially introducing bias into the estimates. Fourth, clinical validation was conducted using an East Asian population, whereas genetic evidence was derived from a multi-ancestral GWAS. This ethnic mismatch may limit the generalizability of our findings and potentially lead to an underestimation of drug effects in populations outside East Asia.

In conclusion, we identified several approved agents as potential treatments for migraine, demonstrating the utility of an integrated approach for drug repositioning. This study also provides insights into the potential mechanisms of action that may be targeted for migraine therapy. By combining GWAS summary statistics, perturbagen data, and real-world claims database, this framework offers time- and cost-efficient strategies for drug repositioning.

## Data Availability

The datasets presented in this study can be found in online repositories. The names of the repository/repositories and accession number(s) can be found below: https://doi.org/10.5281/zenodo.17382613.
