# Supplementary material for: Integrated analysis for drug repositioning in migraine using genetic evidence and claims database
Source: Front Big Data. 2025 Nov 21;8:1677167. doi: 10.3389/fdata.2025.1677167 (PMC12678156; doi:10.3389/fdata.2025.1677167)
Supplement: Supplementary file 1 [file Data_Sheet_1.pdf]

Supplementary Material

1.1 Supplementary Tables

Supplementary Table 1. Definition of the variables

Item	Definition
**Acute migraine drugs**	**WHO-ATC code**
Sumatriptan	N02CC01
Zolmitriptan	N02CC03
Eletriptan	N02CC06
Rizatriptan	N02CC04
Naratriptan	N02CC02
Isopropylantipyrine, Ergotamine tartrate, Anhydrous caffeine	N02CA52
Lasmiditan	N02CC08
**Comorbidities**	**ICD-10 code (Japanese standard disease code)**
Hypertension	I10–I15
Dyslipidemia	E78
Diabetes mellitus	E10–E15
Anxiety disorders	F40–F48
Mood disorders	F30–F39
Epilepsy	G40
Sleep disorders	G47
Malignant neoplasms	C
Chronic kidney disease	N18
Chronic kidney disease stage 5	N185
Rheumatoid arthritis	M05, M060, M068, M069
Other rheumatic diseases	M300, M301, M302, M308, M313, M314, M315, M316, M317, M318 (8845513), M32, M34, M350, M351 (7109008, 7109007), M352, M353, M45
Parkinson's disease	G20
Interstitial pneumonia	J841, J849, M351 (8848245), M051 (8847737)
Peptic ulcer	K25–K27
Gastroesophageal reflux disease	K21
Psoriasis	L40
Behçet's disease	M352
Bronchial asthma	J45
Allergic rhinitis	J30
Overactive bladder	M328 (8843031, 8844583)
**Medical procedures**	**Japanese category code**
Maintainance dialysis	J038, C102, J042
**Preventive Medications**	**WHO-ATC code**
Anti-calcitonin gene-related peptide monoclonal antibodies	N02CD
Antiepileptic drugs (Valproate, topiramate)	N03AG01, N03AX11
Other antiepileptic drugs (Gabapentin, Levetiracetam)	N02BF01, N03AX14
Amitriptyline	N06AA09
Other antidepressants (Nortriptyline, Imipramine, Clomipramine, Trazodone, Mianserin, Fluvoxamine, Paroxetine, Sulpiride, Duloxetine, Fluoxetine)	N06AA10, N06AA02, N06AA04, N06AX05, N06AX03, N06AB08, N06AB05, N05AL01, N06AX21, N06AB03
Beta-blockers	C07
Calcium channel blockers (Lomerizine, Verapamil, Diltiazem, Nicardipine, Flunarizine)	N02CX (2190023), C08DA01, C08DB01, C08CA04, N07CA03
Angiotensin II receptor blockers	C09[CD]
Angiotensin-converting enzyme inhibitors	C09[AB]
**Exposure drugs**	**WHO-ATC code**
Metformin	A10BA
Hypoxia-inducible factor prolyl-hydroxylase inhibitor	B03XA05, B03XA07 ,B03XA08, B03XA09
Folate	B03BB
Rasagiline	N04BD02
Monoamine oxidase inhibitor	N04BD
Sulfonylurea	A10BB
Simvastatin	C10AA01

Statins	C10AA, C10BA05, C10BA06, C10BX03
Nintedanib	L01EX09
Cimetidine	A02BA01
H2-blockers	A02BA
Lamotrigine	N03AX09
Chlorzoxazone	M03BB03
Thiazolidine	A10BG
Apremilast	L04AA32
Leukotriene receptor antagonist	R03DC01, R03DC02, R03DC03
Muscarinic M3 receptor antagonist	G04BD08, G04BD11, G04BD06, G04BD14, G04BD04
Angiotensin receptor neprilysin inhibitor	C09DX04

ATC: Anatomical Therapeutic Chemical; ICD-10: International Classification of Diseases, 10th revision

Supplementary Table 2. Covariate setting for the models in the claims data analysis

Covariate setting
**Common covariates**
Age
Gender
Hypertension
Dyslipidemia
Diabetes
Anxiety disorders
Mood disorders
Epilepsy
Sleep disorders
Malignant neoplasms
Years since the first diagnosis of migraine
Anti-calcitonin gene-related peptide monoclonal antibodies
Antiepileptic drugs
Other antiepileptic drugs
Amitriptyline
Other antidepressants
Beta-blockers
Calcium channel blockers
Angiotensin II receptor blockers
Angiotensin-converting enzyme inhibitors
**Specific covariates for each exposure**
**Metformin**
None
**Hypoxia-inducible factor prolyl-hydroxylase inhibitor**
Chronic kidney disease
Chronic kidney disease stage 5
Maintenance dialysis
**Folate**
Rheumatoid arthritis
Other rheumatic diseases
**Rasagiline**
Parkinson's disease
**Monoamine oxidase inhibitor**
Parkinson's disease
**Sulfonylurea**
None
**Simvastatin**
None
**Statins**
None
**Nintedanib**
Interstitial pneumonia
**Cimetidine**
Peptic ulcer
Gastroesophageal reflux disease
**H2-blockers**
Peptic ulcer
Gastroesophageal reflux disease
**Lamotrigine**
None
**Chlorzoxazone**
None
**Thiazolidine**
None
**Apremilast**

	Psoriasis
	Behçet's disease
**Leukotriene receptor antagonist**	
	Bronchial asthma
	Allergic rhinitis
**Muscarinic M3 receptor antagonist**	
	Overactive bladder
**Angiotensin receptor neprilysin inhibitor**	
	None

Supplementary Table 3. Perturbagens identified through the genetic exploration

GTEx tissue	Cell	Perturbagen name	Rho	P	Perturbagen type†	Description	Candidate approved drug(s)*
artery_aorta	A375.311	MTOR	-0.182	1.12E-06	trt_xpr		**Metformin**
artery_aorta	HA1E	KD-025	-0.180	1.37E-06	trt_cp	ROCK inhibitor	
artery_tibial	ASC	TSPAN4	-0.175	2.05E-06	trt_sh		
colon_sigmoid	HS578T	fostamatinib	-0.176	3.43E-06	trt_cp	Syk inhibitor	
artery_aorta	PC3	G6PD	-0.172	3.78E-06	trt_oe		
prostate	THP1	rolipram	-0.178	4.66E-06	trt_cp	PDE4 inhibitor	**Apremilast**
artery_coronary	MCF7	PCK2	-0.177	6.37E-06	trt_sh		
artery_aorta	MCF7	cercosporin	-0.167	6.68E-06	trt_cp	Photoactivated toxin	
uterus	HA1E	efaproxiral	-0.181	8.36E-06	trt_cp	Oxysensitizer	
whole_blood	LOVO	troglitazone	-0.171	8.73E-06	trt_cp	Thiazolidine	**Thiazolidines**
artery_tibial	NCIH596	tosedostat	-0.163	8.86E-06	trt_cp	Aminopeptidase inhibiitor	
artery_aorta	HA1E	efaproxiral	-0.164	9.67E-06	trt_cp	Antitumor agent, oxysensitizer	
artery_coronary	PC3	BRD-K23488072	-0.173	1.03E-05	trt_cp		
artery_aorta	PC3	carvedilol	-0.163	1.09E-05	trt_cp	beta blocker	
artery_aorta	HEPG2	barasertib-HQPA	-0.163	1.10E-05	trt_cp	Aurora A/B inhibitor	
artery_aorta	ASC.C	IOX2	-0.160	1.54E-05	trt_cp	HIF inhibitor	**HIF-PH inhibitors**
brain_frontal_cortex_ba9	HCC515	EDN1	-0.169	1.63E-05	trt_lig	Endothelin, ligand	
artery_aorta	VCAP	FOLR2	-0.160	1.68E-05	trt_lig		
colon_sigmoid	HEPG2	KLHDC9	-0.162	1.83E-05	trt_sh		
artery_aorta	MCF7	rasagiline	-0.158	1.93E-05	trt_cp	MAO inhibitor	**MAO inhibitors**
artery_aorta	HELA	roscovitine	-0.158	2.10E-05	trt_cp	CDK inhibitor	
artery_aorta	VCAP	STK24	-0.157	2.15E-05	trt_sh		
artery_aorta	HCC515	camptothecin	-0.157	2.30E-05	trt_cp	Topoisomerase inhibitor	
colon_sigmoid	HELA	tramiprosate	-0.159	2.37E-05	trt_cp		
brain_anterior_cingulate_cortex_ba24	RMGI	KO-143	-0.169	2.41E-05	trt_cp	BCRP Inhibitor	
ovary	A375	KD-025	-0.166	2.66E-05	trt_cp	ROCK inhibitor	
whole_blood	VCAP	GADD45A	-0.161	2.75E-05	trt_sh		
brain_nucleus_accumbens_basal_ganglia	A549	XMD-1150	-0.162	3.19E-05	trt_cp		
artery_tibial	MCF10A	GDNF	-0.152	3.35E-05	trt_lig		
brain_nucleus_accumbens_basal_ganglia	HT29	darifenacin	-0.162	3.38E-05	trt_cp	M3 receptor antagonist	**M3 receptor antagonists**
artery_tibial	MDST8	KU-0063794	-0.151	3.47E-05	trt_cp	mTOR inhibitor	**Metformin**
brain_frontal_cortex_ba9	PC3	AZ-628	-0.161	3.61E-05	trt_cp	Raf inhibitor	
artery_tibial	HT29	SD-6-035-A11	-0.151	3.64E-05	trt_cp		
uterus	PC3	cyclobenzaprine	-0.167	3.91E-05	trt_cp	Not in Japan	
ovary	HT29	ATN-161	-0.162	3.98E-05	trt_cp	Integrin inhibitor. A5b1.	

colon_sigmoid	DV90	rotenone	-0.155	4.00E-05	trt_cp	Mitochondria respiration inhibitor	
artery_aorta	HUVEC	AZD-7762	-0.151	4.47E-05	trt_cp	Chk inhibitor	
artery_aorta	HEPG2	REV-5901	-0.151	4.55E-05	trt_cp	lipoxygenase (LOX) inhibitor	**Leukotriene receptor antagonist**
brain_anterior_cingulate_cortex_ba24	VCAP	GPR34	-0.162	4.72E-05	trt_sh		
artery_aorta	MCF7	NTF4	-0.150	4.73E-05	trt_lig		
artery_aorta	SKMEL28	linsitinib	-0.150	4.76E-05	trt_cp	IGF-1 receptor inhbiitor	
artery_aorta	HELA	efaproxiral	-0.150	4.78E-05	trt_cp	Antitumor agent, oxysensitizer	
artery_tibial	PC3	HDAC1-selective	-0.148	4.93E-05	trt_cp	HDAC1 inhibitor	
artery_aorta	HME1	I-BET-151	-0.150	5.14E-05	trt_cp	BET inhibitor	
colon_sigmoid	HCC515	ORM2	-0.152	5.19E-05	trt_lig		
artery_tibial	NPC	glipizide	-0.147	5.35E-05	trt_cp	Sulfonylurea	**Sulfonylureas**
artery_aorta	A375	BRD-K45217009	-0.149	5.35E-05	trt_cp		
artery_coronary	HUVEC	KU-55933	-0.157	5.48E-05	trt_cp	ATM inhibitor	
artery_tibial	FIBRNPC	simvastatin	-0.147	5.52E-05	trt_cp	Statin	**Statins Simvastatin**
artery_aorta	SKMEL28	serdemetan	-0.149	5.65E-05	trt_cp	Antineoplastic agent	
artery_aorta	PC3	sorafenib	-0.148	5.83E-05	trt_cp	Multi-tyrosin kinase inhibitor	**Nintedanib**
artery_tibial	HUH7	BRD-K81826010	-0.146	6.08E-05	trt_cp		
brain_nucleus_accumbens_basal_ganglia	MCF7	olaparib	-0.156	6.14E-05	trt_cp	PARP inhibitor	
artery_aorta	MCF7	camptothecin	-0.148	6.21E-05	trt_cp	Topoisomerase inhibitor	
colon_sigmoid	A375	indacaterol	-0.150	6.30E-05	trt_cp	Long-acting beta agonist	
artery_tibial	NPC	FOXO4	-0.146	6.32E-05	trt_sh		
artery_tibial	HA1E	iso-olomoucine	-0.145	6.93E-05	trt_cp	Cdk5 inhibitor	
brain_nucleus_accumbens_basal_ganglia	DV90	BRD-K98404142	-0.155	6.94E-05	trt_cp		
brain_nucleus_accumbens_basal_ganglia	HA1E	diltiazem	-0.154	7.10E-05	trt_cp	Ca channel blocker	
artery_coronary	MCF7	SB-225002	-0.154	7.17E-05	trt_cp	CXCR2 antagonist	
colon_sigmoid	A549	KLHDC2	-0.149	7.27E-05	trt_sh		
brain_nucleus_accumbens_basal_ganglia	HELA	sacubitril	-0.153	8.07E-05	trt_cp	Neprilysin inhibitor	**Valsartan and sacubitril**
artery_aorta	NCIH1836	rottlerin	-0.145	8.14E-05	trt_cp	PKC inhibitor	
brain_nucleus_accumbens_basal_ganglia	HA1E	CKB	-0.153	8.24E-05	trt_sh	Creatine kinase B	
artery_coronary	HCC515	cimetidine	-0.153	8.38E-05	trt_cp	H2 blocker	**Cimetidine H2 blockers**
artery_aorta	PC3	lamotrigine	-0.145	8.42E-05	trt_cp	Anticonvulsant	**Lamotrigine**
brain_frontal_cortex_ba9	PC3	SLC35F2	-0.153	8.63E-05	trt_sh		
colon_sigmoid	HA1E	MK-0773	-0.147	8.75E-05	trt_cp	Androgen receptor inhibitor	

colon_sigmoid	A549	JUN	-0.147	8.75E-05	trt_sh		
artery_coronary	PC3	chlorzoxazone	-0.152	8.88E-05	trt_cp	MAO inhibitor	**MAO inhibitors**
brain_nucleus_accumbens_basal_ganglia	HCC515	GW-5074	-0.152	8.95E-05	trt_cp	Raf inhibitor	
brain_anterior_cingulate_cortex_ba24	A549	lomerizine	-0.156	8.99E-05	trt_cp	Ca channel blocker	
artery_aorta	HT115	troglitazone	-0.144	9.07E-05	trt_cp	Thiazolidine	**Thiazolidines**
artery_aorta	HEPG2	SB-203580	-0.144	9.08E-05	trt_cp	p38 inhibitor	
artery_coronary	HA1E	fusidic-acid	-0.152	9.09E-05	trt_cp	Steroid antibiotic	
brain_nucleus_accumbens_basal_ganglia	HT29	MCL1	-0.152	9.30E-05	trt_sh		
brain_nucleus_accumbens_basal_ganglia	PC3	candesartan	-0.152	9.30E-05	trt_cp	Angiotensin II receptor blocker	
artery_aorta	MDAMB231	BMS-387032	-0.144	9.36E-05	trt_cp		
artery_coronary	MCF7	BRD-K44833108	-0.152	9.52E-05	trt_cp		
artery_aorta	A549	CTSS	-0.144	9.53E-05	trt_lig		
artery_aorta	A549	fulvestrant	-0.144	9.54E-05	trt_cp	Estrogen receptor inhibitor	
artery_tibial	VCAP	BRD-K56904840	-0.142	9.85E-05	trt_cp		
artery_coronary	A375	TBL3	-0.151	9.91E-05	trt_sh		
brain_nucleus_accumbens_basal_ganglia	SKL.C	PD-0325901	-0.151	9.93E-05	trt_cp	MEK inhibitor	

† trt_cp: Compound; trt_lig: Peptides and other biological agents; trt_sh: shRNA; trt_xpr: CRISPR for loss-of-function

* Except for drugs used for neoplasms and those already included as covariates due to their known therapeutic effects.

Supplementary Table 4. Demographics of the study population in the claims data analysis

**Total, *n***	**214,843**
Age (years),	
median [IQR]	38.0 [26.0, 47.0]
Gender, female (*n*, %)	152661 (71.1%)
Years since the initial diagnosis of migraine	
median [IQR]	0.0 [0.0, 1.6]
Follow-up period (days)	
median [IQR]	965.0 [522.0, 1603.0]
Number of acute migraine medications/year (over time)	
median [IQR]	2 [0, 29]
Number of acute migraine medications/year (first year)	
median [IQR]	10 [5, 40]

IQR: Interquartile range

1.2 RECORD Checklist

The RECORD statement – checklist of items, extended from the STROBE statement, that should be reported in observational studies using routinely collected health data

	Item No.	STROBE items	Location in manuscript where items are reported	RECORD items	Location in manuscript where items are reported
**Title and abstract**					
	1	(a) Indicate the study's design with a commonly used term in the title or the abstract (b) Provide in the abstract an informative and balanced summary of what was done and what was found	Abstract	RECORD 1.1: The type of data used should be specified in the title or abstract. When possible, the name of the databases used should be included.<br><br>RECORD 1.2: If applicable, the geographic region and timeframe within which the study took place should be reported in the title or abstract.<br><br>RECORD 1.3: If linkage between databases was conducted for the study, this should be clearly stated in the title or abstract.	1.1 Abstract<br>1.2 Not applicable but demonstrated in the Materials and methods section<br>1.3 Not applicable
**Introduction**					
Background rationale	2	Explain the scientific background and rationale for the investigation being reported	Introduction (1st and 2nd paragraph)		
Objectives	3	State specific objectives, including any prespecified hypotheses	Introduction (3rd paragraph)		
**Methods**					

Study Design	4	Present key elements of study design early in the paper	Materials and method section ("Validation using a claims database: Study population")	•	•
Setting	5	Describe the setting, locations, and relevant dates, including periods of recruitment, exposure, follow-up, and data collection	Materials and method section ("Validation using a claims database: Study population")		
Participants	6	*(a) Cohort study* - Give the eligibility criteria, and the sources and methods of selection of participants. Describe methods of follow-up<br><br>*Case-control study* - Give the eligibility criteria, and the sources and methods of case ascertainment and control selection. Give the rationale for the choice of cases and controls<br><br>*Cross-sectional study* - Give the eligibility criteria, and the sources and methods of selection of participants<br><br>*(b) Cohort study* - For matched studies, give matching criteria and number of exposed and unexposed<br><br>*Case-control study* - For matched studies, give matching criteria and the number of controls per case	(a) Materials and method section ("Validation using a claims database: Study population")<br><br>(b) Not applicable	RECORD 6.1: The methods of study population selection (such as codes or algorithms used to identify subjects) should be listed in detail. If this is not possible, an explanation should be provided.<br><br>RECORD 6.2: Any validation studies of the codes or algorithms used to select the population should be referenced. If validation was conducted for this study and not published elsewhere, detailed methods and results should be provided.<br><br>RECORD 6.3: If the study involved linkage of databases, consider use of a flow diagram or other graphical display to demonstrate the data linkage process, including the number of individuals with linked data at each stage.	6.1 Materials and method section ("Validation using a claims database: Study population")<br><br>6.2 Materials and method section ("Validation using a claims database: Study population")<br><br>6.3 Not applicable
Variables	7	Clearly define all outcomes, exposures, predictors, potential confounders, and effect modifiers. Give	Materials and method section ("Validation using a claims	RECORD 7.1: A complete list of codes and algorithms used to classify exposures, outcomes, confounders, and effect modifiers should be	Materials and method section ("Validation using a

		diagnostic criteria, if applicable.	database: Variables")	provided. If these cannot be reported, an explanation should be provided.	claims database: Variables"), and Supplementary Table 1 and 2
Data sources/ measurement	8	For each variable of interest, give sources of data and details of methods of assessment (measurement).<br><br>Describe comparability of assessment methods if there is more than one group	Materials and method section ("Validation using a claims database: Study population" and "Validation using a claims database: Variables")		
Bias	9	Describe any efforts to address potential sources of bias	Materials and method section ("Validation using a claims database: Variables")		
Study size	10	Explain how the study size was arrived at	Not applicable		
Quantitative variables	11	Explain how quantitative variables were handled in the analyses. If applicable, describe which groupings were chosen, and why	Materials and method section ("Validation using a claims database: Variables")		
Statistical methods	12	(a) Describe all statistical methods, including those used to control for confounding<br><br>(b) Describe any methods used to examine subgroups and interactions<br><br>(c) Explain how missing data were addressed<br><br>(d) *Cohort study* - If applicable, explain how	(a) Materials and method section ("Validation using a claims database: Statistical analysis")<br><br>(b–e) Not applicable		

		loss to follow-up was addressed<br><br>*Case-control study* - If applicable, explain how matching of cases and controls was addressed<br><br>*Cross-sectional study* - If applicable, describe analytical methods taking account of sampling strategy<br><br>(e) Describe any sensitivity analyses			
Data access and cleaning methods		..		RECORD 12.1: Authors should describe the extent to which the investigators had access to the database population used to create the study population.<br><br>RECORD 12.2: Authors should provide information on the data cleaning methods used in the study.	12.1 Materials and method section ("Validation using a claims database: Study population")<br><br>12.2 Not applicable
Linkage		..		RECORD 12.3: State whether the study included person-level, institutional-level, or other data linkage across two or more databases. The methods of linkage and methods of linkage quality evaluation should be provided.	Not applicable
**Results**					
Participants	13	(a) Report the numbers of individuals at each stage of the study (*e.g.*, numbers potentially eligible, examined for eligibility, confirmed eligible, included in the study, completing follow-up, and analysed)	(a, b) Results section ("Clinical validation of identified drugs using a routinely collected claims database" and Figure 3)	RECORD 13.1: Describe in detail the selection of the persons included in the study (*i.e.,* study population selection) including filtering based on data quality, data availability and linkage. The selection of included persons can be described in the text	13.1 Results section ("Clinical validation of identified drugs using a routinely collected claims

		(b) Give reasons for non-participation at each stage.<br><br>(c) Consider use of a flow diagram		and/or by means of the study flow diagram.	database" and Figure 3)
Descriptive data	14	(a) Give characteristics of study participants (*e.g.*, demographic, clinical, social) and information on exposures and potential confounders<br><br>(b) Indicate the number of participants with missing data for each variable of interest<br><br>(c) *Cohort study* - summarise follow-up time (*e.g.*, average and total amount)	(a, c) Results section ("Clinical validation of identified drugs using a routinely collected claims database" and Supplementary Table 4)<br><br>(b) Not applicable		
Outcome data	15	*Cohort study* - Report numbers of outcome events or summary measures over time<br><br>*Case-control study* - Report numbers in each exposure category, or summary measures of exposure<br><br>*Cross-sectional study* - Report numbers of outcome events or summary measures	Results section ("Clinical validation of identified drugs using a routinely collected claims database" and Supplementary Table 4)		
Main results	16	(a) Give unadjusted estimates and, if applicable, confounder-adjusted estimates and their precision (e.g., 95% confidence interval). Make clear which confounders were adjusted for and why they were included<br><br>(b) Report category boundaries when	(a) Results section ("Clinical validation of identified drugs using a routinely collected claims database" and Figure 3)		

		continuous variables were categorized<br><br>(c) If relevant, consider translating estimates of relative risk into absolute risk for a meaningful time period	(b, c) Not applicable		
Other analyses	17	Report other analyses done—e.g., analyses of subgroups and interactions, and sensitivity analyses	Not applicable		
**Discussion**					
Key results	18	Summarise key results with reference to study objectives	Discussion section (1st paragraph)		
Limitations	19	Discuss limitations of the study, taking into account sources of potential bias or imprecision. Discuss both direction and magnitude of any potential bias	Discussion section (5th paragraph)	RECORD 19.1: Discuss the implications of using data that were not created or collected to answer the specific research question(s). Include discussion of misclassification bias, unmeasured confounding, missing data, and changing eligibility over time, as they pertain to the study being reported.	Discussion section (5th paragraph)
Interpretation	20	Give a cautious overall interpretation of results considering objectives, limitations, multiplicity of analyses, results from similar studies, and other relevant evidence	Discussion section (6th paragraph)		
Generalisability	21	Discuss the generalisability (external validity) of the study results	Discussion section (5th paragraph)		
**Other Information**					
Funding	22	Give the source of funding and the role of the funders for the present study and, if applicable, for the original study on	Funding section		

		which the present article is based			
Accessibility of protocol, raw data, and programming code		..		RECORD 22.1: Authors should provide information on how to access any supplemental information such as the study protocol, raw data, or programming code.	Data Availability Statement section

*Reference: Benchimol EI, Smeeth L, Guttmann A, Harron K, Moher D, Petersen I, Sørensen HT, von Elm E, Langan SM, the RECORD Working Committee. The REporting of studies Conducted using Observational Routinely-collected health Data (RECORD) Statement. *PLoS Medicine* 2015; in press.

1.3 Supplementary explanation of the statistical validation using a claims database

In the validation using a claims database, long-format data were created to account for time-varying variables, as illustrated in the following table for the case of metformin.

ID	time_window	period	metformin	age	gender_female	hypertension	dyslipidemia	diabetes	….	y
1	0	1	0	40	1	0	1	1	…	10
1	1	1	1	41	1	0	1	1	…	5
1	2	0.7	1	42	1	1	1	1	…	20
2	0	1	0	60	0	0	0	0	…	2
2	1	1	0	61	0	0	0	0	…	5
2	2	0.3	0	62	0	0	1	1	…	3
…	…		…	…	…	…	…	…	…	…

ID represents the unique identifier of each patient; time window refers to the number of years in a rolling 1-year window; and period denotes the length of observation for each time window, expressed in years. Each covariate was evaluated based on the information obtained from the exposure and covariate assessment window (Figure 3B). After constructing this data frame, we applied a mixed-effects log-linear model in R (version 4.3.3) using the lmerTest package (version 3.1.3), as illustrated in the following R code:

```
mod <- lmer(log(y + 1) ~ metformin + age + gender_female + hypertension + ….+ (1|ID),
offset=log(period), data=data)
```

After fitting the model, the exponential coefficient $\exp(\alpha)$ was interpreted as the multiplicative effect of the exposure drug on the frequency of prescribed acute migraine medications.
